# Influenza D Virus: The Most Discreet (for the Moment?) of the Influenza Viruses

**DOI:** 10.3390/jcm9082550

**Published:** 2020-08-06

**Authors:** Alessandra Falchi

**Affiliations:** Laboratoire de Virologie, Université de Corse-Inserm, 20250 UR7310 Corte, France; falchi_a@univ-corse.fr

The review of Kumari et al. [1], titled “Emerging Influenza D Virus Threat: What We Know so Far!”, deals with an important issue, although it is not well defined: what we know about the emerging Influenza D virus (IDV). Many congratulations to the authors for putting the focus on such an important and topical subject, but this is not yet fully understood. In this review, the evolution, epidemiology, virology and pathobiology of IDV and the possibility of transmission among various hosts and potential to cause human disease is thoroughly analyzed. Kumari and coworkers, underlined that despite strong results achieved by a considerable number of experimental, epidemiological and serological studies, current knowledge of IDV, a new member of the *Orthomyxoviridae* family, is still limited, especially concerning the evidence that Koch’s postulates are fulfilled for this agent.

This panzootic virus, in multiple animal hosts, was first isolated from a pig with influenza-like illness in 2011 in Oklahoma, USA [2]. Electronic microscopy and real-time reverse transcription-PCR (RT-PCR) revealed that it was neither an influenza A virus (IAV) nor an influenza B virus (IBV). This virus has a genome of seven segments and presented approximately 50% overall homology with influenza C virus (ICV). Antigenic characterization using rabbit polyclonal sera on 10 IDVs from the United States suggested these IDVs can be grouped to two distinct antigenic groups (D/swine/Oklahoma/1334/2011 [D/OK]-like and D/bovine/Oklahoma/660/2013 [D/660]-like influenza viruses) [3]. Serological analyses demonstrated no cross-reactivity with antibodies directed against human IAV, IBV and ICV. Several studies have reported the isolation of IDV in cattle from many geographic areas in different continents (Europe, America and Asia), suggesting that cattle may be a primary natural reservoir for the virus [2,4]. IDV has also been identified in other animal species, such as small ruminants, camelids and horses across countries in different continents (Europe, North America, Africa and Asia), but there is no evidence of infections in chickens and turkeys [5]. Experimental studies in cattle demonstrated that IDV was detected in both lower and upper respiratory tract causing mild signs [6]. However, the coinfection of other respiratory viruses with IDV could cause the bovine respiratory diseases (BRD) which is the most economically significant disease affecting the U.S. cattle industry [7]. A recent study describing multiple years of virological and serological surveillance in the same Mississippi order-buyer cattle facility, reported that 32 IDV isolates with a large extent of phenotypic diversity in replication efficiency and pathogenesis, were recovered from both healthy and sick animals including those with evident antibodies against IDV. Thus, limited protection from pre-existing immunity against IDVs in cattle herds and co-circulation of a wide viral genetic pool could facilitate the high prevalence of IDVs in animal populations [8].

Even if the zoonotic potential of IDV is still unclear and no indications that IDV can cause disease in humans have been found, to date, neither potential threats to exposed individuals nor public health problems can be safely excluded. Indeed, it is important to highlight its ability to replicate and be transmitted among ferrets and guinea pigs, the gold standard for influenza studies animals [9] and its potential to infect a wide number of domestic mammal species. The few seroprevalence studies undertaken to date in humans have shown that the risk of transmission from infected cattle to humans may be very high, with peaks in humans that seem to follow IDV epidemics in animals [10]. Cattle-workers showed seroprevalence rates against IDV above 90%, almost five times higher than the control subjects without contact with cattle [11]. Furthermore, the increased risk of transmission to humans is supported by the finding that the IDV exhibit a broader cellular and host tropism than ICV [2].

What weapons do we have to fight IDV? In terms of strategies to control IDV infection in humans, as explained by the review of Kumari et al. [1], several rapid molecular assays have been evaluated in a short time. These molecular assays ranged from a tetraplex run assay for screening influenza virus types A, B, C and D to one –run RT-PCR assay to simultaneously detect IDV and other 15 respiratory agents [12,13]. Interestingly, a pilot aerosol study, conducting surveillance for human and zoonotic respiratory viruses in an airport setting over a period of nine weeks from January to March 2018 reported molecular evidence of IDV virus and adenovirus in aerosol samples through non-invasive and non-disruptive environmental sampling techniques [14].

Currently, there is no recommended therapy or vaccine available for IDV, and the development of a protective vaccine against IDV have yet to be reported [15,16,17]. Really remarkable the results achieved for a DNA vaccine encoding the consensus hemagglutinin–esterase fusion protein of two lineages of IDV (D/OK and D/660). High-titer neutralizing antibodies against two IDV lineage-representative intranasal infections have been reported for this DNA vaccine tested in a guinea pig model [16].

In conclusion, despite its zoonotic potential to date, the public health significance of IDV appears to be rather low. On the other side, with the increased potential of IDV transmission in and between animal reservoirs, and the high presence of neutralizing antibodies in cattle exposed workers, there is a strong need to evaluate if IDV is zoonotic through One Health surveillance [18]. It is clear that the ability of IDV to cause disease in humans has not been studied in depth and efforts must be relayed in this direction.
